# An automatic fluorescence phenotyping platform to evaluate dynamic infection process of *Tobacco mosaic virus*-green fluorescent protein in tobacco leaves

**DOI:** 10.3389/fpls.2022.968855

**Published:** 2022-09-02

**Authors:** Junli Ye, Jingyan Song, Yuan Gao, Xu Lu, Wenyue Pei, Feng Li, Hui Feng, Wanneng Yang

**Affiliations:** ^1^National Key Laboratory of Crop Genetic Improvement, National Center of Plant Gene Research (Wuhan), Hubei Hongshan Laboratory, Huazhong Agricultural University, Wuhan, China; ^2^Key Laboratory of Horticulture Biology, Ministry of Education, College of Horticulture and Forestry Sciences, Huazhong Agricultural University, Wuhan, China; ^3^Key Laboratory for Quality Regulation of Tropical Horticultural Crops of Hainan Province, College of Horticulture, Hainan University, Haikou, China; ^4^Shenzhen Institute of Nutrition and Health, Huazhong Agricultural University, Wuhan, China

**Keywords:** green fluorescence, digital camera, image processing, tobacco, phenotyping platform

## Abstract

Tobacco is one of the important economic crops all over the world. *Tobacco mosaic virus* (TMV) seriously affects the yield and quality of tobacco leaves. The expression of TMV in tobacco leaves can be analyzed by detecting green fluorescence-related traits after inoculation with the infectious clone of TMV-GFP (Tobacco mosaic virus - green fluorescent protein). However, traditional methods for detecting TMV-GFP are time-consuming and laborious, and mostly require a lot of manual procedures. In this study, we develop a low-cost machine-vision-based phenotyping platform for the automatic evaluation of fluorescence-related traits in tobacco leaf based on digital camera and image processing. A dynamic monitoring experiment lasting 7 days was conducted to evaluate the efficiency of this platform using *Nicotiana tabacum L.* with a total of 14 samples, including the wild-type strain SR1 and 4 mutant lines generated by RNA interference technology. As a result, we found that green fluorescence area and brightness generally showed an increasing trend over time, and the trends were different among these SR1 and 4 mutant lines samples, where the maximum and minimum of green fluorescence area and brightness were mutant-4 and mutant-1 respectively. In conclusion, the platform can full-automatically extract fluorescence-related traits with the advantage of low-cost and high accuracy, which could be used in detecting dynamic changes of TMV-GFP in tobacco leaves.

## Introduction

Tobacco originated in tropical America and spread to Europe, Africa and Asia in the 16th and 17th centuries, which grew to become commercial production crop around the world because of the need of cigarettes and cigars (Arcury and Quandt, 2006; Hanafin and Clancy, 2015). China is the largest tobacco production and consumption in the world (Zhang and Cai, 2003). According to the statistics of the National Bureau of Statistics, the planting area of tobacco in China was 1.981×10^6^hectares, and the yield was 2.156×10^6^ tons in 2020. The total fiscal revenue of tobacco industry was 124.42 billion yuan in 2021, an increase of 3.36%, which positively contributed to the national and local financial income and economic development (China Tobacco, 2022).

*Tobacco mosaic virus* (TMV) is a single-stranded RNA virus that infects lots of plants, including tobacco, other solanaceous and crucifer plants, and several others (Saito et al., 1987; Yamaya et al., 1988; Britt et al., 1998; Summers, 2003; Adeel et al., 2021). According to scientific or economic importance, plant virologists list TMV as one of the top 10 viruses in *Molecular Plant Pathology* (Scholthof et al., 2011). Once the plant is infected with TMV, it would always exist, which makes the plant show symptoms such as deformity of leaves, leaf curling and mottling, dwarfing of plants and stunted growth, leading to yield reduction and economic losses (Ellis et al., 2020; Lv et al., 2020). In addition, TMV seriously affects the quality of tobacco leaves (Lin, 2012). Therefore, the detection of TMV is of great significance to tobacco production.

Traditionally, the main detection methods of tobacco virus are enzyme-linked immuno-sorbent assay (ELISA), electron microscopy (EM), serological identification and Reverse Transcription-Polymerase Chain Reaction (RT-PCR) technology (Hong et al., 1999; Zheng et al., 2011). Van Regenmortel et al. found that ELISA was able to detect a wide spectrum of TMV strains (Vanregenmortel and Burckard, 1980), which is still a common and effective method for virus detection (Lommel et al., 1982; Zhang et al., 2013), and the preparation of antigen by ELISA requires crushing sample tissue to make it fully contact with buffer (Kumar and Prakash, 2016). One of the very first objects that can be observed by EM is TMV (Richert-Poeggeler et al., 2019), then EM became a classical method for studying plant viruses, for example Fang et al. combined electron microscopy negative staining with ultrathin sectioning and DAS (Double antibody sandwich)-ELISA to detect virus in tobacco shred (Fang et al., 2008). Due to its convenient, high sensitivity and specificity, RT-PCR is widely used in tobacco virus detection, especially multiplex RT-PCR (Letscher et al., 2002; Yang et al., 2010; Dai et al., 2012; Yang Y. et al., 2020). However, RT-PCR requires going through the steps of primer designing, total RNA extraction, cDNA synthesis, PCR reaction and product electrophoresis analysis, among which the total RNA extraction needs to grind the tobacco leaves (Song et al., 2007; Kumar et al., 2011; Zhao et al., 2015). It’s not difficult to find that these methods are destructive, laborious to operate, and some require expensive reagents. Therefore, a non-destructive method that can automatically detect tobacco virus has become expected.

In recent years, spectroscopic techniques have become potential methods for non-destructive detection of plant diseases, especially hyperspectral imaging technology (Sankaran et al., 2010; Thomas et al., 2018). Combined with successive projections algorithm (SPA) and machine-learning, Zhu et al. confirmed that hyperspectral imaging could be used for distinguishing TMV-infected tobacco leaves from healthy samples (Zhu et al., 2017). Gu et al. detected the infection of *Tomato spotted wilt virus* (TSMV) non-destructively in tobacco leaves at an early stage using hyperspectral imaging technology, and found that the NIR region (780–1,000 nm) is important for TSMV detection (Gu et al., 2019). Polder et al. developed a method for the detection of *Potato virus Y* (PVY) infected potato plants based on hyperspectral image data and fully convolutional neural network (CNN), which showed that the recall values were slightly lower than the accuracy of crop expert (Polder et al., 2019). Nagasubramanian et al. identified charcoal rot disease in soybean stems using 3D deep CNN model based on hyperspectral data, for which classification accuracy was 95.73% (Nagasubramanian et al., 2019). Therefore, hyperspectral imaging plays an important role in non-destructive detection of plant diseases. However, the high cost of devices is one of the important limitation factors for the usage of hyperspectral imaging technology (Bhagwat and Dandawate, 2021). Consequently, low-cost and non-destructive detection technology is the current research direction.

Markers are commonly used as auxiliary tools in biological research. Green fluorescent protein (GFP), which absorbs blue light and emits green fluorescence, is an important marker for gene expression and protein localization and was widely used in the observation of plant virus proliferation (Chalfie et al., 1994; Huang et al., 1997; Tsien, 1998; Phillips, 2001; Teuscher and Ewald, 2018). An advantage of GFP is that the protein is stable and usually has no toxicity to living cells, so when it is connected to plant virus by transgenic technology, and then the virus is inoculated into plants, the expression of the virus in plants can be seen by tracking the green fluorescence under blue light or ultraviolet (UV) light (Chalfie, 2009; Ma, 2009). UV light can stimulate blue light (Ma, 2009), which is why researchers use UV light to observe green fluorescence. At present, there are many plant viruses that were observed for their expression in plants by GFP, such as TMV, *Tomato spotted wilt virus*, *Plantago asiatica mosaic virus*, *potato virus X*, *Wheat dwarf virus*, *Lettuce necrotic yellows virus*, *Rice grassy stunt virus*, *Rice ragged stunt virus*, *Tomato bushy stunt virus* and so on (De Ronde et al., 2013; Minato et al., 2014; Wang et al., 2014). Thus, GFP is a mature marker for studying plant virus expression, and was used to monitor the infection of TMV on tobacco leaves in this study.

The methods of GFP detection include fluorescence microscopy (Yuste, 2005; Torrado et al., 2008; Mann et al., 2015), fluorescence spectrometer (Richards et al., 2003), portable handheld UV light with camera (Harper et al., 1999; Saxena et al., 2011; Shamekova et al., 2014), electronic microscopy (Liu et al., 2003; Adeel et al., 2021), and hyperspectral imaging system (Annamdevula et al., 2013). However, the cost of microscope device and its maintenance is relatively high for fluorescence microscope (Luby-Phelps et al., 2003; Toman, 2004). And most fluorescence spectrometers and EM are lab-based, high-cost, and not suitable for high throughput detection of GFP, while sample observation by portable handheld UV light with camera depends on artificial (Saxena et al., 2011). Therefore, it is of great significance to develop a low-cost machine for automatic evaluation green fluorescence of GFP. With the development of digital imaging techniques, we can get plant traits by using RGB imaging, which has become an important component of high-throughput plant phenotyping platforms (Yang W. et al., 2020). High-throughput plant phenotyping platforms can quickly obtain various morphological and physiological traits information of a large number of plants, and complete a large number of phenotyping traits measurement in the shortest time (Fahlgren et al., 2015). In fact, these platforms provide a new way for GFP detection, which will promote the development of TMV expression research in tobacco leaves.

The objective of this study is to develop a low-cost and automatic phenotyping platform for monitoring GFP expression in tobacco leaves change over time and automatically evaluating fluorescence-related traits, then analyzing the dynamic infection of TMV in tobacco leaves. Given its measuring efficiency and relatively low hardware cost, RGB digital imaging was preferred in this study. To achieve the goal, this study applied machine vision and automatic control technology to improve inspection accuracy and efficiency of this phenotyping platform.

## Materials and methods

### Materials

The tobacco varieties used in this study were *Nicotiana tabacum L.*, with one wild-type strain SR1, and four mutants generated by RNA interference technology. These four mutants had different resistance to TMV, which could be detected by the platform. With a total of 14 samples, mutant-1 had two biological repeats, while SR1 and other 3 mutant lines had three biological repeats. All of these SR1 and 4 RNAi lines were cultivated by the steps in Supplementary Table 1.

### Transient expression in *Nicotiana tabacum L.* leaves

*Agrobacterium* (with TMV-GFP plasmid) was activated in LB medium (with 100 μg/mL spec + 10 μg/mL rif) at 28°C, 200 r/min for 16 h. The activated Agrobacterium was added to the induction LB medium (with 100 μg/mL spec, 10 μg/mL rif, 40 μM acetosyringone, 10 μM pH 5.6 morpholine ethanesulfonic acid) at a ratio of 1:50 and incubated at 28°C, 200 r/min for 16–20 h. The induced Agrobacterium was centrifuged at 3,000 *g*/min for 5 min, the supernatant was poured off, and the bottom bacteria were collected. The bacteria were dispersed with resuspension medium (H_2_O + 10 M MgCl_2_ + 40 μM acetosyringone) and diluted to OD600 = 0.1. The diluted Agrobacterium was placed at room temperature for 2∼4 h. Agrobacterium was injected from the abaxial surface of the leaves into 4-week-old *Nicotiana tabacum L*. with a 1 ml syringe without needle until the whole leaf was infiltrated with the bacterial solution. The injected tobacco was placed in an incubator at 24°C during the day and 20°C at night, with 12 h of light per day, and the expression of GFP in the tobacco could be detected after 3∼7 days.

**FIGURE 1:**
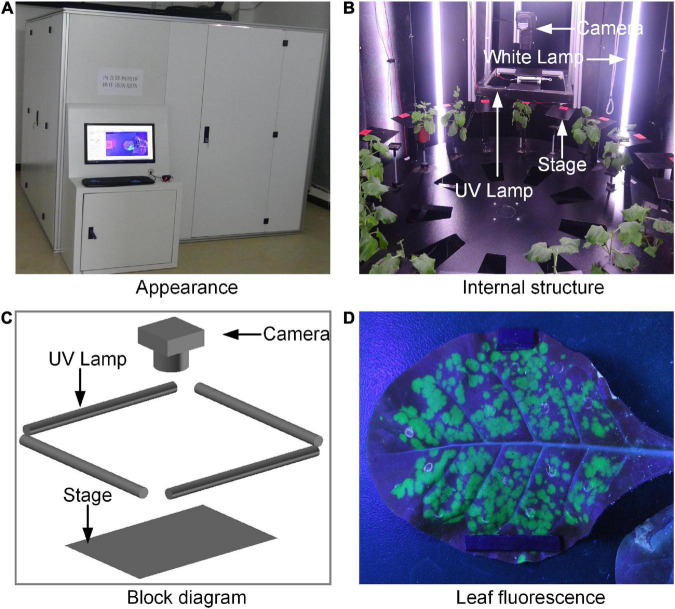
The prototype of fluorescence phenotyping platform: **(A)** Appearance of the platform, **(B)** the inter structure of the platform, **(C)** the block diagram of the acquisition part, and **(D)** the acquired leaf fluorescence image.

Two days after inoculation, the plants were moved to the platform for experiment lasting 7 days, and images were automatically collected every 1 h. Every inoculated leaf was fixed on the stage with small magnets, and the images of tobacco leaf inoculated with TMV-GFP were shown as Figure 1D.

### System description

The prototype of the platform was shown in Figures 1A,B, and the acquisition part was shown in Figure 1C. The platform consisted of four UV lamps, 12 white lamps, a digital camera (Nikon, D90, Japan) equipped with a focus lens of 35 mm, a rotation stage, a working stage with 18 small leaf stages and 18 small pot pallets, a programmable logic controller (PLC, CP1H-Y20DTD, Omron, Japan), a computer workstation, and a darkroom. Every small leaf stage had its own unique digital tag. The object distance was 28 cm. The UV lamps were turned on during image acquisition, while the white lamps were turned off, so that the images of tobacco leaves with clear green fluorescence could be captured. As soon as the images were collected, the UV lamps were turned off and the white lamps were turned on for the tobacco growing. The opening and closing time of the white lamps would be set according to the normal sunrise and sunset. With the working stage as the reference, the leaf stages were fixed, and the pot pallets could move up and down. The UV lamps, white lamps, and the rotation stage were connected to the PLC. The PLC and digital camera were connected to the computer workstation. All the stages were black for the convenience of segmentation of images. The system was placed in the darkroom for stable imaging environment. In order to observe the interior of the darkroom conveniently, four sides of the darkroom can be opened.

### System control

The system control software for the platform was developed by LabVIEW 8.6 (National Instruments, United States). In the software, the automatic observation and resulting images for each leaf were displayed and stored systematically according to current time string. In addition, the PLC was programmed by CX-Programmer 7.3 (Omron, Japan). The control of the digital camera was programmed in the C++ language and compiled into a dynamic link library for LabVIEW 8.6 calling.

**FIGURE 2:**
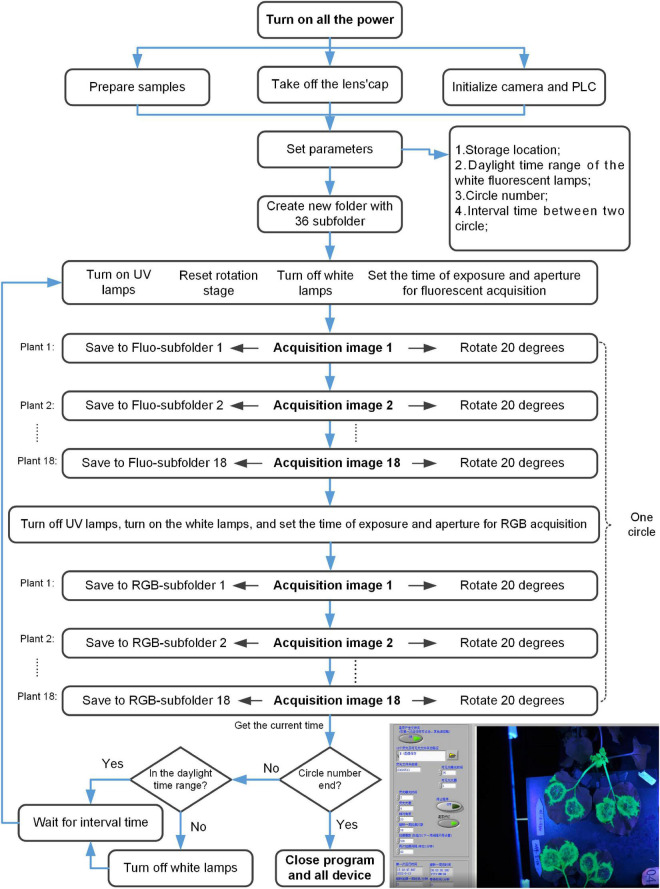
Diagram of control and data acquisition for the fluorescence phenotyping platform.

**FIGURE 3:**
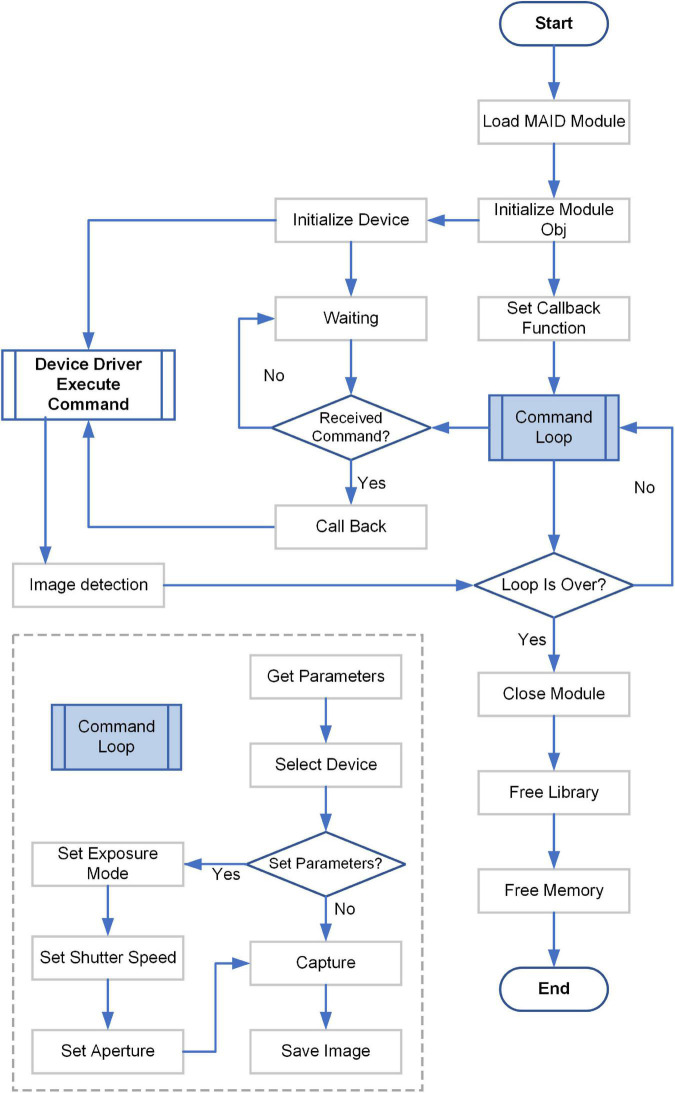
Control diagram for the digital camera.

The system control flowchart included the following steps (Figure 2; Supplementary Videos 1, 2). (1) Turn on all the power, prepare samples, and take off the lens’ cap of the digital camera. The program began to initialize the digital camera and PLC. (2) Set the following parameters, including storage location, daylight time range of the white fluorescent lamps, circle number, and the interval time between two circles. And a new folder with 36 subfolders would be created in the storage location. (3) Turn off the white lamps, turn on the UV lamps, reset the rotation stage, and set the time of exposure and aperture for fluorescent acquisition automatically. (4) Acquire fluorescent image (The diagram of control for the digital camera was shown in Figure 3). The rotation stage rotated 360 degrees, a total of 18 fluorescent images were collected and saved to corresponding Fluo-subfolder. (5) Turn off the UV lamps, turn on the white lamps, and set the time of exposure and aperture for RGB acquisition automatically. (6) Acquire RGB image (The diagram of control for the digital camera was shown in Figure 3). The rotation stage rotated 360 degrees, a total of 18 RGB images were collected and saved to corresponding RGB-subfolder. The acquisition of one circle was ended. (7) The program determined whether the circle number was ended. If it was, the program ended. Otherwise, the program would proceed with the following steps. (8) The program automatically got the current time and determined whether it was in the daylight time range. If it was, the program would turn on the white lamps. Otherwise, the program would turn off the white lamps. (9) Wait for the interval time between two circles. In this process, the program would continue to obtain the current time and repeated step (8). If the waiting was finished, the program would go to step (3).

Next, the control for the digital camera would be introduced in detail (Figure 3). These contents corresponded to the acquire image part of the upper part. Each acquire image step in the Figure 2 would goes through all the steps in Figure 3. The detailed flowchart included the following steps. (1) Load the *MAID Module* of the digital camera. (2) Initialize the *Module Obj* of the digital camera. The *MAID Module* was downloaded from the official website of digital camera. (3) Initialize the digital camera, and waiting for the command. (4) Set the callback function. (5) Go on the command loop. The commands included the following contents: get parameters from the earlier step [Figure 2 step (2)], select device, check if set parameters or not, capture image and save image. (6) Execute command from step (5). (7) Check if the computer detected the image. If no, just waiting. If yes, go on the next step. (8) Check if the command loop is over. If no, go on the step (5). If yes, go on the next step. (9) Close the Module, and free the Library and memory.

### Image process

**FIGURE 4:**
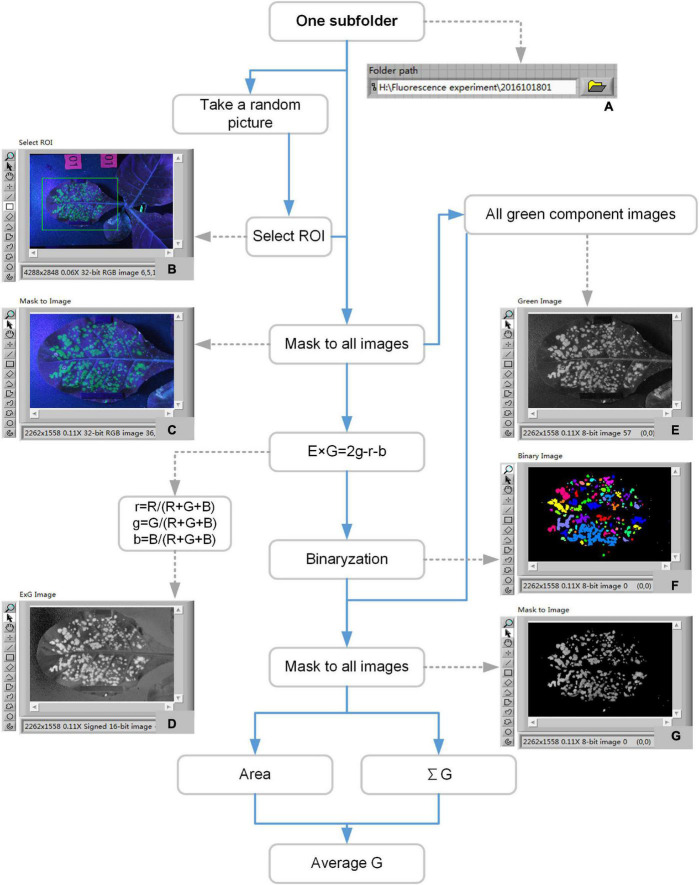
Flow chart of image processing and data analysis to extract fluorescence-related traits. Panel **(A)** was the currently processed folder path. Panel **(B)** was the original image. Panel **(C)** was the cropped image. Panel **(D)** was the ExG image. Panel **(E)** was the green channel image. Panel **(F)** was the binary pseudo-color image. Panel **(G)** was the green channel image of the binary area.

All the image processing software was developed in LabVIEW 8.6. The flow chart of image processing and data analysis was shown in Figure 4. After a round of 7 days experiment, 36 subfolders corresponding to 18 samples were got. The fluorescent image processing and data analysis included the following steps. (1) Choose one subfolder path (Figure 4A). (2) The program randomly selected an image to display on the front panel for the selecting of region of interest (ROI). Then a ROI would be got (Figure 4B). (3) Cut all the images of the subfolder according to the ROI (Figure 4C). (4) Convert all the images to ExG images according to the formula (Figure 4D). (5) Binary option with the ExG images. The results were binary images (Figure 4F). (6) Extract green channel images from the RGB images of step (3) (Figure 4E). (7) Mask the binary images (Figure 4F) to green channel images (Figure 4E). The results were the green channel images of the binary area (Figure 4G). (8) Calculate the parameters related to area, brightness, and change trend according to the images of Figure 4G.


(1) A⁢r⁢e⁢a=∑w

(2) $$B\,r\,i\,g\,h\,t\,n\,e\,s\,s=\frac{\sum q}{\text{Area}}$$

where w indicates the fluorescence pixel number, where q indicates the gray value of the fluorescence pixel.

## Results and discussion

### Performance of the platform

**FIGURE 5:**
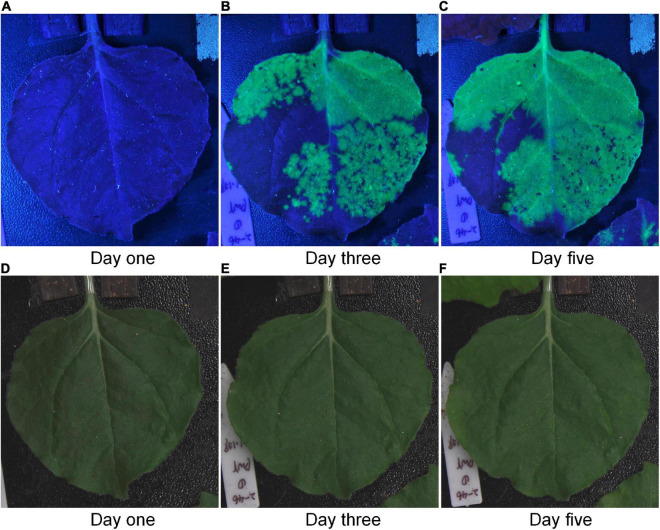
The comparison of fluorescent images and RGB images of the same tobacco leaf. Panels **(A–C)** were fluorescent images. Panels **(D–F)** were RGB images.

To evaluate the fluorescence-related traits in tobacco leaf, we successfully developed a low-cost machine-vision-based phenotyping platform. To test the performance of this platform, we compared the fluorescent images with the RGB images collected by the system. The comparison results of the same leaf were shown in Figure 5 (Supplementary Video 3), including the obtained fluorescent and RGB images at the first day, the third day, and the fifth day, respectively. It could be seen intuitively that fluorescent images taken by this platform showed great differences, while there was no difference between the RGB images. This result indicated that the platform could detect the changes of green fluorescence in tobacco leaves through fluorescent images. Therefore, through this system, we can use cheap and simple equipment to observe the change of green fluorescence in tobacco leaves which was invisible by human eyes. Furthermore, this system can also be used to explore biological applications. For example, researchers have already used the system and achieved some preliminary results. Zhang et al. successfully observed the difference in the effect of artificial small RNA with only one nucleotide difference on plant disease resistance using the platform developed in this study (Zhang et al., 2020). And Zhang et al. accurately identified the antiviral ability differences of antiviral compounds with different subtle chemical modifications in plants (Zhang et al., 2021). These results showed that this system had potential to be widely used in plant research.

### Fluorescence image to reflect dynamic infection process of *Tobacco mosaic virus*-green fluorescent protein

**FIGURE 6:**
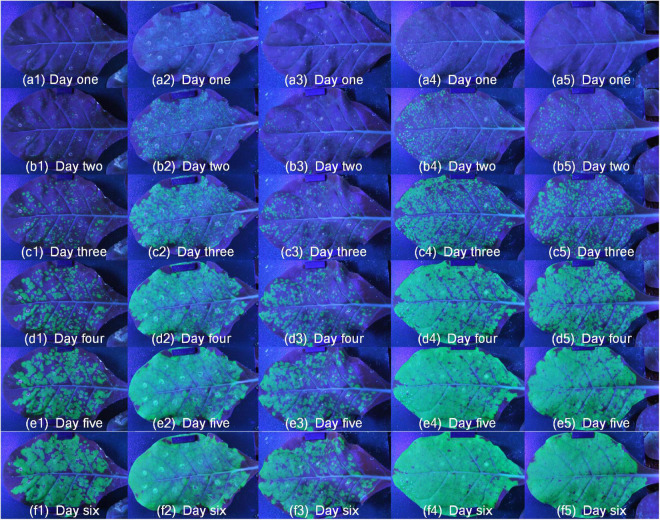
The change of green fluorescence of SR1 and 4 mutant lines. Numbers 1 to 5 represented mutant-1, SR1, mutant-3, mutant-2 and mutant-4, respectively, and panels **(a–f)** represented different periods over time.

The images of dynamic fluorescence changes were collected 2 days after inoculation with TMV-GFP. All samples were moved to the platform, and the system began to automatically capture fluorescent images. After image acquisition, take the steps described in the section “Image process” for image processing. Fist, the fluorescent images of ROI were extracted. Figure 6 showed the green fluorescence contrast of SR1 and 4 mutant lines samples (Supplementary Video 4), each row represented the same growth period, and each column represented different periods of the same sample. It could be seen that the leaves of SR1 and 4 mutant lines had different green fluorescence area, brightness and rates of green fluorescence change. Furthermore, it was potential to analyze the ability of TMV infection by analyzing the ratio of green fluorescence area to total leaf area. For these SR1 and 4 mutant lines samples, green fluorescence changed rapidly from c to e, and then changed slowly. It also showed that the green fluorescence area and brightness of mutant-1 was minimum, which indicated that the infection ability of TMV in mutant-1 was the weakest compared with SR1 and 4 mutant lines. In addition, it was potential to analyze the difference of TMV infection process by analyzing the results of different samples in the same period.

**FIGURE 7:**
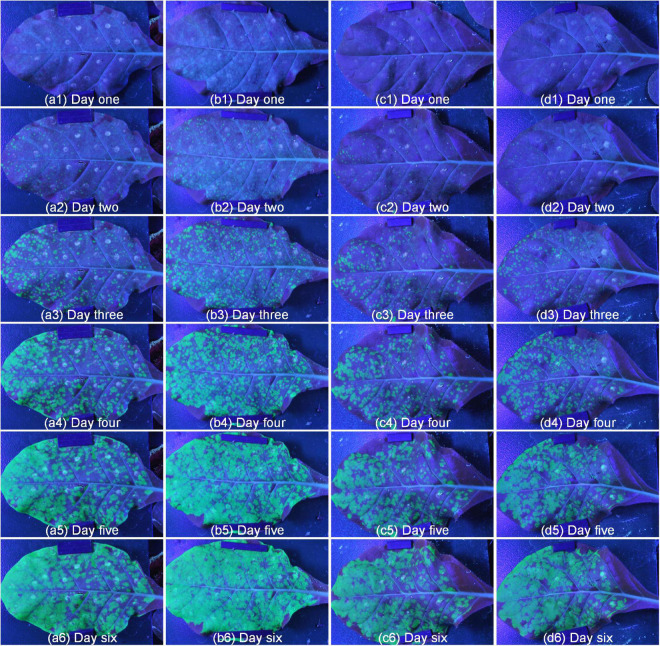
The repeatability results of two different lines. Panels **(a,b)** represented different repetitions of the same line, panels **(c,d)** represented different repetitions of another line, number 1 to 6 represented different periods.

To test the performance of the system, we conducted repeated experiments and compared the results between the repeated experiments. As shown in Figure 7, there were two different lines, where a and b were the same line, c and d were another line, and 1 to 6 represented different growth periods. Comparing with the same line, the green fluorescence area and brightness had similar trends in the same period, which proved the stability of the system. In addition, the green fluorescence area and brightness of SR1 and 4 mutant lines were tested by independent samples Kruskal-Wallis test using SPSS 26.0, and the results of paired comparison were shown in Tables 1, 2 where each sample included all the data of its biological repeats. The results showed that the distribution of green fluorescence area and brightness between SR1 and other 4 mutant lines had extremely significant differences.

**TABLE 1:** The independent samples Kruskal–Wallis test of green fluorescence area.

Sample 1 & sample 2	Test statistics	Standard error	Standard test statistics	Sig.	Adj. Sig.
mutant-1 & mutant-2	–285.375	43.194	–6.606861	0.000	0.000
mutant-1 & mutant-3	192.046	43.194	4.446164	0.000	0.000
mutant-1 & mutant-4	–225.662	43.194	–5.224416	0.000	0.000
mutant-1 & SR1	–131.377	43.194	–3.041582	0.002	0.024
mutant-2 & mutant-3	477.421	38.634	12.357659	0.000	0.000
mutant-2 & mutant-4	59.713	38.634	1.545621	0.122	1
mutant-2 & SR1	153.998	38.634	3.986104	0.000	0.001
mutant-3 & mutant-4	–417.708	38.634	–10.812038	0.000	0.000
mutant-3 & SR1	–323.423	38.634	–8.371555	0.000	0.000
mutant-4 & SR1	94.285	38.634	2.440483	0.015	0.147

Each line tested the null hypothesis that the distribution of sample 1 was the same as that of sample 2. It showed progressive significance (bilateral test). The significance level was 0.05. Adj. Sig.: Significance values had been adjusted by Bonferroni correction method for multiple tests.

**TABLE 2:** The independent samples Kruskal–Wallis test of green fluorescence brightness.

Sample 1 & sample 2	Test statistics	Standard error	Standard test statistics	Sig.	Adj. Sig.
mutant-1 & mutant-2	–454.167	43.194	–10.514654	0.000	0.000
mutant-1 & mutant-3	30.709	43.194	0.710966	0.477	1.000
mutant-1 & mutant-4	–686.714	43.194	–15.898484	0.000	0.000
mutant-1 & SR1	–554.704	43.194	–12.842256	0.000	0.000
mutant-2 & mutant-3	484.876	38.634	12.550625	0.000	0.000
mutant-2 & mutant-4	–232.547	38.634	–6.019305	0.000	0.000
mutant-2 & SR1	–100.538	38.634	–2.602338	0.009	0.093
mutant-3 & mutant-4	–717.423	38.634	–18.569930	0.000	0.000
mutant-3 & SR1	–585.414	38.634	–15.152963	0.000	0.000
mutant-4 & SR1	132.010	38.634	3.416966	0.001	0.006

Each line tested the null hypothesis that the distribution of sample 1 was the same as that of sample 2. It showed progressive significance (bilateral test). The significance level was 0.05. Adj. Sig.: Significance values had been adjusted by Bonferroni correction method for multiple tests.

**FIGURE 8:**
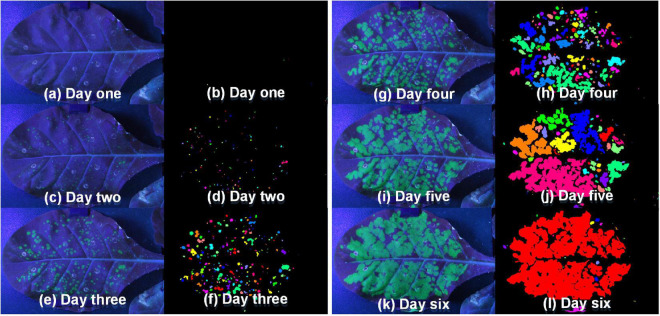
The fluorescent images [panels **(a,c,e,g,i,k)**] taken by this platform and binary pseudo-color images [panels **(b,d,f,h,j,l)**] of tobacco leaf in the different periods (Day one to Day six).

After confirming the feasibility and stability of the system, the fluorescent images taken by this platform were further processed to obtain binary images with the green channel. Figure 8 showed the fluorescent images and binary pseudo-color images of tobacco leaf at different periods. It could be seen that the green fluorescence area was gradually becoming larger with the growth of the tobacco plant. More importantly, it could also show that the segmentation method used in this paper can effectively segment the fluorescent part from the whole leaf.

### Fluorescence-related traits extraction

**FIGURE 9:**
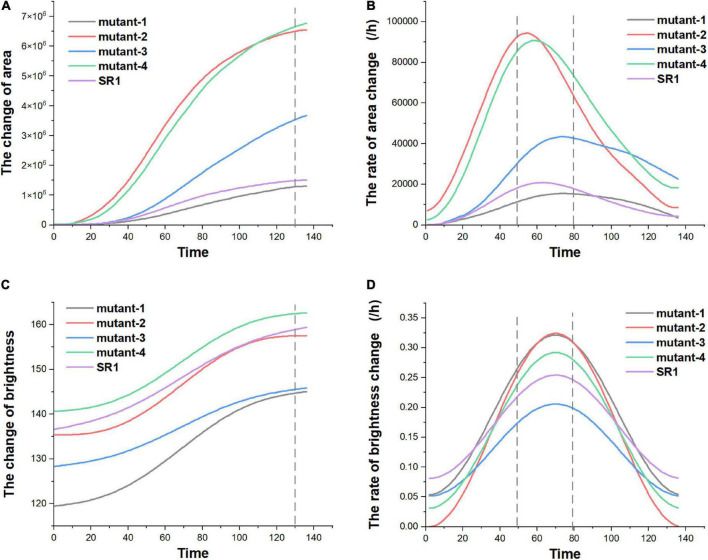
The green fluorescence area and brightness change over time of SR1 and 4 mutant lines plants. Panel **(A)** showed the change of green fluorescence area, panel **(B)** showed the rate of green fluorescence area change per hour, panel **(C)** showed the change of green fluorescence brightness, and panel **(D)** showed the rate of green fluorescence brightness change per hour.

In order to analyze the change of green fluorescence more vividly, the parameters related to area, brightness, and change trend were calculated according to binary pseudo-color images, and the change curves were made. Pre-processing by Savitzky–Golay convolution smooth and Fast Fourier Transform filter in Origin 2022, the variation trends of green fluorescence area and brightness of these SR1 and 4 mutant lines samples (taking the average of biological repeats) were shown in Figure 9 (made by Origin 2022). According to Figure 9, we could see that the green fluorescence area in tobacco leaves gradually increased with the image acquisition time, and the rate of green fluorescence area change reached the maximum in 50–80 h (the dotted lines in Figure 9B), then the rate gradually slowed down. After 130 h (the dotted line in Figure 9A), the green fluorescence areas were basically stable and reached the maximum, and the order of the maximum was: mutant-4, mutant-2, mutant-3, SR1, and mutant-1. Besides, the maximum green fluorescence area and the peak rate of green fluorescence area change of mutant-4 and mutant-2 were much larger than SR1 (shown in Table 3). It showed that after stabilization, the order of maximum green fluorescence brightness was: mutant-4, SR1, mutant-2, mutant-3, and mutant-1, and the changes of green fluorescence brightness of these SR1 and 4 mutant lines samples had similar trend (shown in Figure 9C). Consistent with the change of green fluorescence area, the green fluorescence brightness tended to be stable after 130 h (the dotted line in Figure 9C) of acquisition, and the peak rate of green fluorescence brightness happened in the range of 50–80 h (the dotted line in Figure 9D). From Table 3, we could also see that there was little difference in the peak rate of green fluorescence brightness change per hour among SR1 and 4 mutant lines samples, which indicated that green fluorescence brightness changed slowly per hour. Therefore, we could quickly analyze the micro unquantifiable process through the macroscopic observation by this platform.

**TABLE 3:** The peak and median of the rate of green fluorescence area and brightness change per hour.

	The rate of area change		The rate of brightness change	
		
	Peak	Median	Peak	Median
mutant-1	15910.94	9424.22	0.32133	0.13390
mutant-2	95554.74	47778.06	0.32414	0.16213
mutant-3	44325.49	26276.06	0.20574	0.077195
mutant-4	91594.69	49013.85	0.29196	0.13055
SR1	21317.21	10978.22	0.25334	0.090165

### Advantage and limitation

Based on the above results, the platform developed in this study could identify green fluorescence in tobacco leaves and realize the non-destructive detection of TMV-GFP. And the platform had been applied to explore the molecular mechanism of tobacco disease resistance through the expression of tobacco disease-GFP in tobacco leaves (Zhang et al., 2020, 2021). According to the knowledge we know, in order to reflect the characteristics of the platform developed in this study, it was compared with the other GFP detection methods, including fluorescence microscope, fluorescence spectrometer, portable handheld UV light with camera, electron microscope and hyperspectral imaging, mainly from five aspects: damage, cost, detection speed, automaticity and whether the results were visualized, and the results were shown in Supplementary Table 2.

Compared with other methods that much depends on artificial, such as EM and portable handheld UV light with camera, the platform developed in this study was highly-automated (Saxena et al., 2011; Adeel et al., 2021). Without making slices or grinding samples, the platform was non-destructive for samples. At the same time, automatic detection saved the time of preparing reagents and making slices, which made the platform realize rapid detection (Liu et al., 2003). More importantly, the platform developed in this study was low-cost with nearly $7,600. Compared with the fluorescence detection system and instruments on the market, such as chlorophyll fluorescence imaging system, fluorescence microscopes/spectrometer, EM and hyperspectral camera, the cost of this platform was greatly reduced about 6∼26 times. In addition, the platform could also realize the visualization of GFP distribution in tobacco leaves (shown in Figure 8), which made it easy to observe the movement of TMV in tobacco leaves only through the visual images. Therefore, the platform is a good method for detecting TMV-GFP.

However, this study still requires improvement. Firstly, the number of samples could be extended to hundreds of samples in future research to fit the high-throughput detection requirement. The current platform could only control temperature. In future research, the control units of environmental factors such as humidity can be added to better simulate the growth environment of tobacco during shooting and monitoring fluorescence signals. And the platform can also be built in the specified environment according to the needs of the researchers, which makes its application scope wider. Besides, the fluorescence-related traits extracted in this study included green fluorescence area, green fluorescence brightness, the change rate of green fluorescence area and brightness, and green fluorescence area ratio. In the future, 3D imaging and microscopic imaging techniques could be combined to mine the deep information of fluorescence-related traits to better qualitatively or quantitatively observe the infection of TMV-GFP in tobacco leaves. Moreover, only the infection of TMV-GFP in tobacco leaves was detected in this study, and the system has the potential to the infection of other plant viruses in stem, spike and other parts of the plant. Furthermore, the fluorescence-related traits could be combined with other omics to analyze the genetic mechanism behind them, and then provide new ideas for tobacco disease resistance breeding. In a word, the platform developed in this study has made some achievements in the research of low-cost, non-destructive and automatic detection of plant virus.

### Further application perspective

In this study, we observed the movement of fluorescence signals in different tobacco leaves at seeding stage through the infection of TMV-GFP, and the movement rate was quantified by calculating fluorescence-related traits. In addition, when comparing the small differences in early fluorescence movement of different tobacco leaves, the platform could quantify them with data. In the future, we can try to visualize signal molecules in tobacco leaves to observe the direction and speed of signal transmission. And the following four points are the focus of our future research based on the platform:

1. Screening of transgenic plants: in plant transgenic experiments, multiple transgenic plants with different insertion sites and different insertion copies can be obtained by an *Agrobacterium* infection (Filipenko et al., 2009; Vain and Thole, 2009). We can identify the differences of resistance or growth and development speed between different plants by the platform, and quickly find suitable plants.
2. Study on circadian rhythm of biological clock: plants have circadian rhythms (McClung, 2006). The characteristics of the platform make it contributes to observe and analyze the circadian rhythms of plant development and resistance during its diurnal change.
3. Accurate identification of efficacy of new pesticides: in the development of pesticides, different molecular modifications may cause subtle differences in pesticide efficacy. The platform can be used to accurately and quantitatively analyze the efficacy of different pesticides to identify the most suitable products (Zhang et al., 2021).
4. Monitoring of plant endogenous substances: adding visual molecular tags is a useful way to observe the endogenous substances in plants (Greer and Szalay, 2002). Through adding visual molecular tags, we can observe the synthesis and transportation of these endogenous substances in plants for a long time by the platform.

## Conclusion

This paper described an automatic fluorescence phenotyping platform to evaluate the fluorescence-related traits in tobacco leaf, including area, brightness and change trend. Based on image processing and data analysis, the infection of TMV-GFP in tobacco leaves can be displayed accurately and real-timely by digital camera, which can further deduce the TMV resistance of different tobacco samples objectively and efficiently. In summary, the low-cost and automatic platform that provides a novel method for the study of plant inoculated with virus through phenotypic information could be applied to the study of tobacco disease resistance breeding, including identification of resistance genes and screening of transgenic disease-resistant plants.

## Data availability statement

The raw data supporting the conclusions of this article will be made available by the authors, without undue reservation.

## Author contributions

HF and JY designed the research, performed the experiments, analyzed the data, and wrote the manuscript. JS and YG analyzed the data and wrote the manuscript. XL and WP performed the experiments. FL provided the tobacco samples and also performed experiments. WY supervised the project, designed the research, and wrote the manuscript. All authors contributed to the article and approved the submitted version.

## Supplementary material

Supplementary Video 1 Operation procedure of the automatic fluorescence phenotyping platform.

Supplementary Video 2 The acquisition process of software interface.

Supplementary Video 3 The comparison of fluorescent images and RGB images of the same tobacco leaf.

Supplementary Video 4 The change of green fluorescence of SR1 and 4 mutant lines.
